# Prevalence of low back pain and associated risk factors amongst adult patients presenting to a Nigerian family practice clinic, a hospital-based study

**DOI:** 10.4102/phcfm.v5i1.441

**Published:** 2013-05-15

**Authors:** Adetola M. Ogunbode, Lawrence A. Adebusoye, Temitope O. Alonge

**Affiliations:** 1 Family Medicine Department, University College Hospital, Ibadan, Nigeria; 2 Department of Orthopaedics and Trauma, University College Hospital, Ibadan, Nigeria

## Abstract

**Background:**

Low back pain (LBP) is a common health problem with concomitant disability which has assumed a public health importance in our setting.

**Objectives:**

The aim of this study was to determine the prevalence of LBP and associated risk factors amongst adult patients attending the General Outpatients’ Clinic of the University College Hospital in Ibadan, Nigeria.

**Method:**

This was a cross-sectional study of 485 respondents. A semi-structured questionnaire was used to obtain information on socio-demography, lifestyle, occupation and other risk factors associated with LBP.

**Results:**

There were 288 (59.4%) female and 197 (40.6%) male respondents. The point prevalence of LBP was 46.8%. Occupational activities, previous back injury and tobacco smoking were significant associated factors for the total population. For the female respondents, logistic regression analysis showed that a waist circumference of 88 cm or more, dysmenorrhea, previous back injury and being engaged in an occupation were the most significant factors associated with LBP. However, previous back injury was the most significant factor associated with LBP for the male respondents.

**Conclusion:**

The prevalence of LBP amongst adult patients in our setting is high, with preventable and treatable predisposing factors. Public health efforts should be directed at educating people on occupational activities and lifestyle habits.

## Abstrait

**Prévalence des lombalgies et facteurs de risque associés chez les patients adultes se présentant dans un centre médical familial du Nigeria, étude en milieu hospitalier**

**Contexte:**

La lombalgie est un problème de santé courant, associé à un handicap, ce problème étant important sur le plan de la santé publique dans notre contexte.

**Objectifs:**

L'objectif de cette étude était de déterminer la prévalence des lombalgies et des facteurs de risque associés chez les patients adultes se présentant au centre de consultation externe de l'hôpital universitaire d'Ibadan, au Nigeria.

**Méthode:**

Il s'agit d'une étude transversale réalisée auprès de 485 personnes interrogées. Un questionnaire semi-structuré a été utilisé afin d'obtenir des informations sur les facteurs sociodémographiques, le style de vie, la profession ainsi que d'autres facteurs de risque associés à la lombalgie.

**Résultats:**

Le groupe de personnes interrogées se composait de 288 (59,4%) femmes et de 197 (40,6%) hommes. La prévalence ponctuelle des lombalgies était de 46,8%. Les activités professionnelles, des blessures dorsales antérieures et la consommation de tabac étaient des facteurs associés significatifs pour la population totale. Pour les femmes interrogées, l'analyse de régression logistique a montré qu'un tour de taille de 88 cm ou plus, une dysménorrhée, des blessures dorsales antérieures et l'activité professionnelle étaient les facteurs associés à la lombalgie les plus significatifs. Chez les hommes interrogés, les blessures dorsales antérieures constituaient le facteur le plus important associé à la lombalgie.

**Conclusion:**

La prévalence de la lombalgie chez les patients adultes est élevée dans notre contexte, les facteurs de prédisposition pouvant faire l'objet d'une prévention et d'un traitement. Les efforts de santé publique devraient s'attacher à éduquer la population sur les activités professionnelles et les habitudes de vie.

## Introduction

Musculoskeletal problems are prevalent amongst the adult population.^1^ Globally, chronic low back pain (LBP) is a common health problem.^2^ People with chronic LBP experience huge social, mental, physical and occupational disruptions.^3^ The mental impact of LBP include anxiety, depression and sleeplessness, whilst poor physical performance and deterioration in health status are the physical impacts.^1^ LBP results in an inability to carry out social activities and it decreases the capability to perform occupational activities since it mostly affects adults of working age.^1, 4^ Disability caused by LBP stems from the pain and/or loss of function inflicted on the sufferers. Chronic LBP is one of the four disabilities causing musculoskeletal conditions – the others being osteoarthritis, osteoporosis and rheumatoid arthritis.^5^ The economic burden of LBP on the society, especially in low-resourced continents like Africa, is enormous and continues to increase. Billions of dollars spent annually on managing LBP further constrains the fragile health care system in Africa, which is already ravaged by the HIV epidemic.^5^

About 40% of sick absences from work is because of LBP - making it the second most common cause of workplace absenteeism after the common cold.^6^ Chronic LBP is the most common musculoskeletal problem encountered in the workplace, with attendant loss of quality of life and financial difficulty.^7^ Chronic LBP is prevalent in many industrialised societies with prevalence rates of 21% and 39% being reported in the general population - and even higher in the occupational setting.^8^ In the USA, LBP is the commonest musculoskeletal illness with 12% – 30% of the population affected at any given time.^9^

Occupation-related factors are the most important risks associated with LBP.^9^ More than 80% of the population will experience an episode of LBP at some time during their lives.^10^ The clinical course is benign for most, with 95% of those afflicted recovering within a few months of onset. Some, however, will not recover and will develop chronic LBP, i.e. pain that lasts for three months or longer.^10^

Risk factors for developing LBP could be immutable (non-modifiable) or mutable (modifiable). The immutable factors are age, parity, previous history of LBP and major scoliosis, whilst the mutable factors include a sedentary lifestyle, obesity, tobacco smoking and drug dependence.^11^ Other mutable factors are occupation-related: poor posturing, prolonged sitting, twisting, bending, stooping and lifting of heavy loads.^11^

There is scanty information available on LBP in resource-constrained countries like Nigeria.^7^ This may be due to fact that LBP is perceived to be of little public health importance when compared with other medical conditions such as hypertension and diabetes mellitus.^7^ This study was carried out to determine the point prevalence and risk factors of LBP amongst adult patients presenting to a family practice clinic in Nigeria.

## Ethical consideration

The head of the Family Medicine department (UCH, Ibadan) approved this study. Respondents gave their informed consent before the administration of their questionnaire and physical examination.

## Methods

### Context of the study

The study was conducted at the General Outpatients’ (GOP) Clinic of the Family Medicine Department of the University College Hospital (UCH) in Ibadan, Nigeria. UCH is a tertiary institution founded in 1957 in the cosmopolitan city of Ibadan. Ibadan is the capital of the Oyo state in south-western Nigeria and has a population of 3.6 million people, mainly of the Yoruba tribe.^12^ Most patients coming to UCH are seen first at the GOP Clinic, which functions as a secondary care clinic within a tertiary hospital setting. Patients are at first contact seen by consultants and resident doctors in family medicine.

### Design

This was a cross-sectional study.

### Study population

The population consisted of 485 patients who presented to the GOP Clinic (UCH) between 15 January 2011 and 30 March 2011. The inclusion criteria were all patients aged 18 years and above who consented to take part in the study. Those who did not consent to the study, who had congenital deformity of the spine or who were too ill to participate in the study were excluded.

### Sampling technique

All adult patients who met the study criteria were consecutively selected.

### Procedure

A semi-structured questionnaire was used to interview the respondents. The questionnaire was pretested to determine if the questions were clear and comprehensive enough to address the set objectives of the study and necessary amendments were then made. Information obtained from the respondents included their socio-demographic characteristics and lifestyle habits. Additionally, their health care utilisation pattern was obtained by asking them about the number of times they had been hospitalised for LBP, the total period spent on admission, the frequency of outpatient hospital visits in the last year and the treatment they sought for their LBP. Anthropometric measurements of height, weight, waist and hip circumferences were carried out for all respondents.

The weight (in kilogram) was divided by the height (meter) and then squared to calculate the body mass index (BMI). The BMI was graded along the World Health Organization's (WHO) classification, where ‘Underweight’ refers to a BMI less than 18.5 kg/m^2^, ‘Normal’ to a BMI of 18.5 kg/m^2^ – 24.9 kg/m^2^, ‘Overweight’ to a BMI of 25.0 kg/m^2^ – 29.9 kg/m^2^ and ‘Obese’ to a BMI greater than 30.0 kg/m^2^. To identify individuals with possible health risks, the waist circumference of the respondents was measured and this was based on the threshold values of ≥ 88 cm for women and ≥ 102 cm for men.^14^ The waist/hip ratio (WHR) was calculated by dividing the waist circumference by the hip circumference.^14^ The cut-off point of WHR was defined as ≥ 0.85 for women and ≥ 1.00 for men.^14^ The questionnaire took an average of 22 min to be completed.

### Anthropometric measurements

Height was recorded to the nearest 0.1 m with a measurement stand (stadiometre) that was positioned on a flat surface. The respondents were asked to remove their shoes and their heels were positioned against the wall, with their scapula, buttocks and heels resting against the wall. Female respondents were asked to remove their headwear. The weight of the respondents was measured to the nearest 0.1 kg using a standard weighing scale manufactured by Hana, China. Respondents stood on the weighing scale after removing their personal effects. The researcher made the readings whilst standing in front of the respondents and the zero mark was checked for accuracy after every reading. Waist and hip circumferences were measured to the nearest 0.1 m using a flexible non-elastic measuring tape.

#### Follow-up

Respondents were treated for their primary health complaints and those needing further treatment were referred to other specialty clinics within the facility.

### Analysis

Administered questionnaires were checked, sorted and coded serially at the end of each study day. The data was entered, cleansed and analysed using SPSS^®^ (version 16). Descriptive statistics was employed for the socio-demography, lifestyle and health care utilisation pattern of the respondents. Categorical and discrete variables were tested using the Chi-square statistics and continuous variables were tested using the *t*-test. The relationship between socio-demography, lifestyle, health care utilisation and LBP was explored using logistic regression analysis. The *p-*value of significance was set at ≤ 0.05.

## Results

**FIGURE 1:**
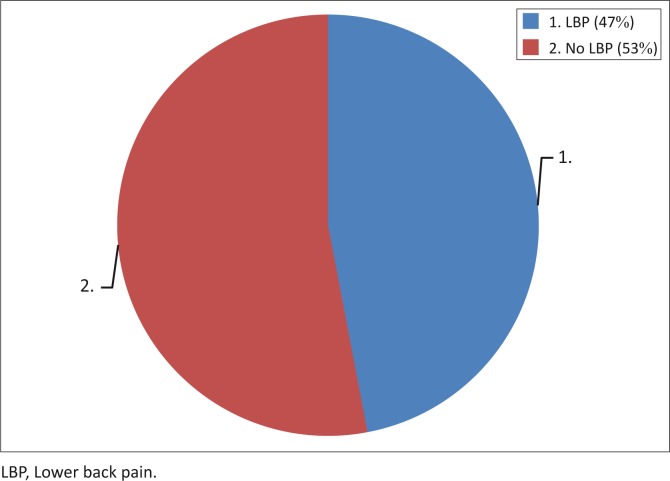
Point prevalence of low back pain.

There were 288 (59.4%) female and 197 (40.6%) male respondents in the study population, with the female to male ratio being 1.5 to 1. The mean ± standard deviation (SD) of the respondents’ age was 42.5 ± 15.5 years (range: 18 to 85 years). Their monthly income ranged from 2000 to 400 000 Naira ($13.33 to $2666.67) with a median income of 18 500 Naira ($123.33). The modal age group was less than 30 years and only 70 respondents (14.4%) were elderly. The majority of the respondents were married and 306 (63.1%) were literate, having completed the first 10 years of formal education (i.e. attained senior secondary and tertiary education). There were 331 respondents (68.2%) living with their immediate family (spouse and children or grandchildren) and 363 (74.8%) were currently engaged in occupational activities. The male respondents were mostly self-supporting whereas the females were mostly dependent on their spouses for financial support (Table 1). The self-reported point prevalence of LBP was 46.8% (Figure 1).

**TABLE 1:** Socio-demographic characteristics of the respondent.

Variables	Characteristics	Female		Male		Total	
		
		*n* = 288	%	*n* = 197	%	*N* = 485	%
Age group (years)	≤ 30	86	62.3	52	37.7	138	100.0
	31– 40	74	64.9	40	35.1	114	100.0
	41–50	58	58.0	42	42.0	100	100.0
	51– 60	38	60.3	25	39.7	63	100.0
	> 60	29	41.4	41	58.6	70	100.0
Marital status	Single	54	47.8	59	52.2	113	100.0
	Married	196	59.4	134	40.6	330	100.0
	Separated and/or divorced	7	77.8	2	22.2	9	100.0
	Widowed	31	93.9	2	6.1	33	100.0
Educational level attained	No education	42	62.7	25	37.3	67	100.0
	Primary school	55	62.5	33	37.5	88	100.0
	Junior secondary school	14	58.3	10	41.7	24	100.0
	Senior secondary school	53	47.7	58	52.3	111	100.0
	Tertiary	124	63.6	71	36.4	195	100.0
Living arrangement	Alone	26	32.9	53	67.1	79	100.0
	Spouse	167	60.5	109	39.5	276	100.0
	Children and/or grandchildren	46	83.6	9	16.4	55	100.0
	Relations	47	69.1	21	30.9	68	100.0
	Friends	2	28.6	5	71.4	7	100.0
Number of children	1 –2	81	54.0	69	46.0	150	100.0
	3 –4	57	60.6	37	39.4	94	100.0
	≥ 5	86	65.2	46	34.8	132	100.0
	1 –2	64	58.7	45	41.3	109	100.0
Financial support	Self	99	42.7	133	57.3	232	100.0
	Spouse	110	87.3	16	12.7	126	100.0
	Parent	44	60.3	29	39.7	73	100.0
	Children and/or grandchildren	27	64.3	15	35.7	42	100.0
	Friends and other relatives	8	69.2	4	30.8	12	100.0
Occupational status	Presently engaged in an occupation	214	59.0	149	41.0	363	100.0
	Not engaged in an occupation	74	60.7	48	39.3	122	100.0
Social class	I	1	20.0	4	80.0	5	100.0
	II	8	57.1	6	42.9	14	100.0
	III	53	52.0	49	48.0	102	100.0
	IV	16	64.0	9	36.0	25	100.0
	V	210	61.9	129	38.1	339	100.0

**FIGURE 2:**
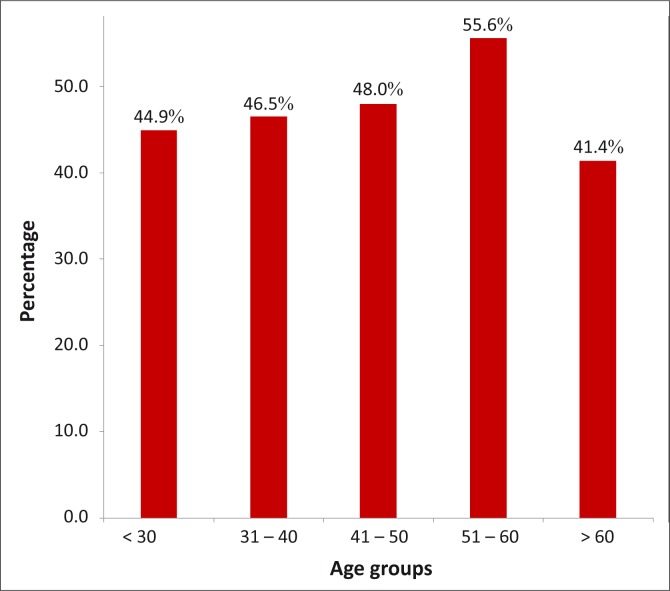
Prevalence of low back pain by age group.

Table 2 shows the socio-demographic characteristics of the respondents by the prevalence of LBP. The point prevalence of LBP was higher amongst men compared with women (50.3% vs. 44.4%), but without statistical significance (χ^2^ = 1.586, *p* = 0.208). The prevalence of LBP increased gradually with age: from 44.9% for respondents younger than 30 years to 55.6% for the age group 51 years - 60 years. However, a reduction in the prevalence of LBP was observed after the age of 60 (Figure 2). There was therefore no statistical association between the prevalence of LBP and age (χ^2^ = 3.007, *p* = 0.558). A higher prevalence of LBP was found in respondents who only had primary school education (55.7%), lived alone (50.6%), had more than five children (52.3%) and were self-supporting (49.6%), as shown in Table 2. The prevalence of LBP was significantly higher amongst respondents who were currently engaged in occupational activities than those who were not (49.9% vs. 37.7%), where χ^2^ = 5.421 and *p* = 0.020 (Table 2). The majority (80%) of the respondents in social class I had LBP. However, Table 2 shows that there was no significant association between social class and LBP (χ^2^ = 4.185, *p* = 0.382).

**TABLE 2:** Socio-demographic characteristics of the respondents by the prevalence of low back pain.

Variables	Characteristics	LBP		No LBP		Total		*χ*^2^	*df*	*P*
		
		*n* = 227	%	*n* = 258	%	*N* = 485	%			
Age group (years)	≤ 30	62	44.9	76	55.1	138	100.0	3.007	4	0.557
	31–40	53	46.5	61	53.5	114	100.0			
	41–50	48	48.0	52	52.0	100	100.0			
	51–60	35	55.6	28	44.4	63	100.0			
	> 60	29	41.4	41	58.6	70	100.0			
Sex	Female	128	44.4	160	55.6	288	100.0	1.586	1	0.208
	Male	99	50.3	98	49.7	197	100.0			
Marital status	Single	56	49.6	57	50.4	113	100.0	5.756	3	0.124
	Married	157	47.6	173	52.4	330	100.0			
	Separated and/or divorced	5	55.6	4	44.4	9	100.0			
	Widowed	9	27.3	24	72.7	33	100.0			
Educational level attained	No education	28	41.8	39	58.2	67	100.0	3.724	4	0.445
	Primary school	49	55.7	39	44.3	88	100.0			
	Junior secondary school	11	45.8	13	54.2	24	100.0			
	Senior secondary school	51	45.9	60	54.1	111	100.0			
	Tertiary	88	45.1	107	54.9	195	100.0			
Living arrangement	Alone	40	50.6	39	49.4	79	100.0	1.060	4	0.901
	Spouse	129	46.7	147	53.3	276	100.0			
	Children and/or Grandchildren	23	41.8	32	58.2	55	100.0			
	Relations	32	47.1	36	52.9	68	100.0			
	Friends	3	42.9	4	57.1	7	100.0			
Number of children	None	71	47.3	79	52.7	150	100.0	2.202	3	0.532
	01–02	42	44.7	52	55.3	94	100.0			
	03–04	57	43.2	75	56.8	132	100.0			
	≥ 5	57	52.3	52	47.7	109	100.0			
Financial support	Self	115	49.6	117	50.4	232	100.0	2.605	4	0.626
	Spouse	60	47.6	66	52.4	126	100.0			
	Parent	29	39.7	44	60.3	73	100.0			
	Children and/or grandchildren	18	42.9	24	57.1	42	100.0			
	Friends and other relatives	5	41.7	7	58.3	12				
	Self	115	49.6	117	50.4	232	100.0			
Occupational status	Presently engaged in an occupation	181	49.9	182	50.1	363	100.0	5.421	1	0.020†
	Not engaged in an occupation	46	37.7	76	62.3	122	100.0			
		1	20.0	5	100.0	4	0.382			
Social class	I	4	80.0	1	20.0	5	100.0	4.185	4	0.382
	II	4	28.6	10	71.4	14	100.0			
	III	48	47.1	54	52.9	102	100.0			
	IV	11	44.0	14	56.0	25	100.0			
	V	160	47.2	179	52.8	339	100.0			

LBP, Low back pain.

† Significant at 5% level of significance.

The highest proportion of LBP (63.6%) was found amongst respondents who commonly adopted the stooping position during their daily activities, whilst the lowest proportion (48.5%) was found in those who commonly adopted the standing position (Table 3). There was no significant association between commonly adopted posture and LBP (χ^2^ = 2.174, *p* = 0.704). The prevalence of LBP was significantly higher amongst respondents who had previous back injury (91.3%) compared with those who did not (44.8%), where χ^2^ = 14.64 and *p* < 0.001. The lifestyle habits of the respondents showed that 71 respondents (14.6%) regularly consumed alcohol, 12 respondents (2.5%) smoked tobacco and 102 respondents (21.0%) engaged in regular physical exercise. The prevalence of LBP was significantly higher in respondents who smoked tobacco than those who did not (91.7% vs. 45.7%), where χ^2^ = 9.946 and *p* = 0.002. However, no association was found between the prevalence of LBP and alcohol consumption or physical exercise. Mattress or foam (94.8%) was the commonest sleeping material used by the respondents, with the majority (83.7%) using the soft type. The majority of respondents using either the firm type (55.4%) or the orthopedic type (70.0%) had LBP. However, there was no significant association between the sleeping material and LBP (χ^2^ = 1.950, *p* = 0.377). Amongst the female respondents, the prevalence of LBP was significantly higher in those who had dysmenorrhea compared with those who did not (57.9% vs. 39.6%), where χ^2^ = 7.565 and *p* = 0.006 (Table 3).

**TABLE 3:** Lifestyle habits of the respondents by the prevalence of low back pain.

Habits	Characteristics	LBP		No LBP		Total		*χ*^2^	*df*	*P*
		
		*n* = 227	%	*n* = 258	%	*N* = 485	%			
Posture mostly adopted during daily activities	Standing	94	48.5	100	51.5	194	100	2.174	4	0.704
	Sitting	68	50.7	66	49.3	134	100			
	Stooping	7	63.6	4	36.4	11	100			
	Squatting	4	57.1	3	42.9	7	100			
Previous back injury	Yes	21	91.3	4	8.7	25	100	14.647	1	0.001†
	No	206	44.8	254	55.2	460	100			
Alcohol	Yes	30	42.3	41	57.7	71	100	0.692	1	0.406
	No	197	47.6	217	52.4	414	100			
Tobacco	Yes	11	91.7	1	8.3	12	100	9.946	1	0.002†
	No	216	45.7	257	54.3	473	100			
Regular physical exercise	Yes	49	48	53	52	102	100	0.079	1	0.779
	No	178	46.5	205	53.5	383	100			
Sleeping material	Mattress/foam	213	46.3	247	53.7	460	100	1.950	2	0.377
	Bare floor	6	46.2	7	53.8	13	100			
	Mat	8	66.7	4	33.3	12	100			
Type of mattress‡	Soft	170	44.2	215	55.8	385	100	5.129	2	= 0.077
	Firm	36	55.4	29	44.5	65	100			
	Orthopaedic	7	70	3	30	10	100			

LBP, Low back pain.

† Significant at 5% level of significance.

‡ Type of mattress did not add up to 485 because it was a subset of sleeping material.

Anthropometric measurements of the respondents showed that the mean weight of the respondents was 66.7 kg ± 13.8 kg (37 kg – 129 kg) and the mean height was 1.63 m± 0.09 m (range: 1.10 m –1.90 m). The mean body mass index (BMI) was 25.2 kg/m^2^ ± 5.5 kg/m^2^ (16.2 kg/m^2^ – 51.9 kg/m^2^). The mean weight of male respondents (67.3 kg ± 1.3 kg) was more than those of the females (66.4 kg ± 1.4 kg) without a significant difference (*t* = 0.539, *p* = 0.463). The mean height of the male respondents was significantly higher than those of their female counterparts (1.67 m ± 0.09 m vs. 1.60 m ± 0.08 m), where *t* = 83.340 and *p* < 0.0001. Conversely, the mean BMI of the female respondents was significantly higher than those of their male counterparts (25.91 ± 5.52 vs. 24.16 ± 5.29), where *t* = 11.758 and *p* = 0.001. The mean waist circumference of the respondents was 79.2 cm ± 12.9 cm (50 cm – 128 cm) and the mean hip circumference was 87.2 cm ± 15.4 cm (43 cm – 133.1 cm). The mean waist/hip ratio (WHR) was 0.91 ± 0.07 (0.48 – 1.36). The mean waist and hip circumference of the female respondents was significantly higher than those of their male counterparts (81.11 ± 13.79 vs. 76.37 ± 10.89, where *t* = 16.001 and *p* < 0.0001, and 89.82 ± 16.10 vs. 83.29 ± 13.45, where *t* = 21.473, *p* < 0.0001) respectively. However, the mean WHR of the females was significantly lower than those of the males (0.908 ± 0.07 vs. 0.923 ± 0.08), where *t* = 5.267 and *p* = 0.022 (Table 4).

**TABLE 4:** Physical characteristics of the respondents by the prevalence of low back pain.

Characteristics	Gender	Sub-characteristics	LBP		No LBP		Total		*χ*^*2*^	*df*	*P*
		
			*n* = 227	%	*n* = 258	%	*N* = 485	%			
BMI	-	Underweight	16	41.0	23	59.0	39	100.0	0.739	3	0.864
	-	Normal	103	47.0	116	53.0	219	100.0			
	-	Overweight	69	48.6	73	51.4	142	100.0			
	-	Obese	39	45.9	46	54.1	85	100.0			
Waist circumference (cm)	Males	< 102	92	49.2	95	50.8	187	100.0	1.643	1	0.200
		≥ 102	7	70.0	3	30.0	10	100.0			
	Females	< 88	81	38.2	131	61.8	212	100.0	12.656	1	0.001†
		≥ 88	47	61.8	29	38.2	76	100.0			
Waist/hip ratio	Males	< 1.00	93	50.5	91	49.5	184	100.0	0.094	1	0.760
		≥ 1.00	6	46.2	7	53.8	13	100.0			
	Females	< 0.85	20	42.6	27	57.4	47	100.0	0.081	1	0.775
		≥ 0.85	108	44.8	133	55.2	241	100.0			

LBP, Low back pain; BMI, Body Mass Index.

† Significant at 5% level of significance.

Table 5 depicts the physical characteristics of the respondents by the prevalence of LBP. The majority of the respondents (45.2%) had a normal BMI, whilst 46.8% were either overweight or obese. The highest proportion of LBP (48.6%) was found in those who were overweight, but there was no significant association between LBP and BMI (χ^2^ = 0.739, *p* = 0.864). A higher proportion of men (70%) with a waist circumference of 102 cm or more reported LBP than those with a waist circumference less than 102 cm (49.2%). However, this difference was not statistically significant (χ^2^ = 1.643, *p* = 0.200). Amongst the females, the prevalence of LBP was significantly higher in those with a waist circumference of 88 cm or more than in those whose waist circumference was less than 88 cm (61.8% vs. 38.2%), where χ^2^ = 12.656 and *p* < 0.001. Additionally, women with a WHR of 0.85 or more had a higher prevalence for LBP than those with a WHR less than 0.85 (44.8% vs. 42.6%), where χ^2^ = 0.081 and *p* = 0.775.

**TABLE 5:** Logistic regression of significant risk factors and low back pain.

Variables	Characteristics	β	Sig.	Odds Ratio Exp(β)	95.0% C.I. for Exp(β)	

					Lower	Upper
Females	Waist circumference	0.029	0.005†	1.029	1.009	1.05
	Consumes tobacco	21.538	1	2.26E + 09	0	-
	Dysmenorrhea	0.816	0.012†	2.26	1.197	4.268
	Previous back injury	2.335	0.045†	10.333	1.048	101.875
	Engaged in occupation	0.87	0.011†	2.388	1.219	4.676
	Constant	-44.911	1	0	-	-
Males	Consumes tobacco	-21.501	0.999	0	0	-
	Previous back injury	2.871	0.006†	17.662	2.256	138.265
	Engaged in occupation	0.073	0.839	1.076	0.531	2.177
	Constant	42.638	0.999	3.29E + 18	-	-
	Engaged in occupation	0.073	0.839	1.076	0.531	2.177
	Constant	42.638	0.999	3.29E + 18	-	-

† significant at 5% level of significance.

Of the 227 respondents who had LBP, only a few (22.5%) reported experiencing their first episode of LBP. The average duration of the LBP was 24 months and 6 respondents (2.6%) had been admitted to hospital for LBP. The average duration of hospitalisation was 3 days. Sixty-three respondents (27.8%) with LBP reported visiting the hospital on an outpatient basis 2.1 ± 1.8 times in the last year. Of these 63 respondents, 26 (41.3%) visited the hospital on an outpatient basis once, 27 (42.9%) visited twice, 5 (7.9%) visited three times and 5 (7.9%) visited four or more times in the last year.

Logistic regression tests were used separately for female and male respondents using all the variables that showed significant association with LBP. For the female respondents, waist circumference of ≥ 88 cm (*p* = 0.005, OR = 1.029, CI =1.009 – 1.050), dysmenorrhea (*p* = 0.012, OR = 2.260, CI = 1.197 – 4.268), previous back injury (*p* = 0.045, OR = 10.333, CI = 1.048 – 101.875) and being engaged in an occupation (*p* = 0.011, OR = 2.388, CI = 1.219 – 4.676) were found to be most associated with LBP. Amongst the male respondents, previous back injury (*p* = 0.006, OR = 17.662, CI = 2.256 – 138.265) was found to be most associated with LBP (Table 5).

## Discussion

There is a global burden of LBP. The prevalence amongst Africans is also rising and it is a cause for concern.^5^ The prevalence of LBP in this study was found to be 46.8%. This is similar to the point prevalence of LBP amongst rice farmers in Thailand, a developing country.^15^ It is also comparable to the prevalence of 46% in a study amongst staff in a rural hospital in Nigeria,^8^ but it is higher than the point prevalence of 20% from a study amongst office workers.

The prevalence of LBP in this study was higher amongst men compared with women. In a review by Punnett, the attributable factor for LBP was also higher amongst men (41%) than women (32%).^2^ The reason proffered was that men usually engage in occupations associated with heavy physical workload and whole-body-vibration compared with women.^2^ This higher prevalence in males was corroborated by another group of researchers.^7^ In contrast to the findings in our study, female rice farmers in Thailand were more likely to develop LBP than males.^15^ Another study amongst staff in a rural hospital also found that female workers had a greater prevalence of LBP.^8^

Some authors reported that the risk of developing LBP increases with advancing age and amongst females, however, others studies reported no such association between these factors.^16^ Louw reported in a systematic review that the point prevalence of LBP amongst African adolescents and adults was 12% and 32% respectively.^5^ Similarly, Taechasubamorn et al. reported the highest prevalence of LBP amongst young adults aged 25 years to 34 years.^15^ Older people were found to experience the more persistent and severe form of LBP compared with young adults.^17^ A report from Sweden showed that LBP was common amongst elderly Post Office pensioners who had previously engaged in lifting heavy loads for more than 20 years.17

In this study, occupational activities and previous back injury were significantly associated with LBP. Thirty-seven percent of back pain worldwide accounts for an estimated 0.8 million disability-adjusted life years (DALY) with resultant work time and economic loss.^18^ Physical efforts, such as manual exertion and exposure to whole-body-vibration are the common physical ergonomics related to LBP.^9^ Some authors found that the majority of those reporting LBP were frequently exposed to ‘stressful situations’ in their occupation.^11^

The highest proportion of LBP in this study was found amongst respondents who commonly adopted the stooping position during their daily activities. This is comparable to the findings of another Nigerian study that associated LBP with heavy physical work, bending, poor posture and prolonged sitting or standing.^8^ In addition, most rice farmers experienced increased LBP from slouched sitting (56.2%), forward bending (70.8%) and lifting (83.2%).^15^

The majority (57.3%) of the respondents using either the firm or orthopedic mattresses had LBP. Our study found no significant association between LBP and the sleeping materials (*p* = 0.377). This result differed from the multicenter trial by Kovacs, where pain and disability amongst patients with chronic, non-specific LBP improved when using the medium firm mattresses.^19^ The firmness of the mattress was rated in their study along the European Committee for Standardization scale, with the firmest mattress rated as 1 and the softest rated as 10.^19^ Self-assessment of the mattress type was carried out by the respondents in this study, which could have affected the results.

Tobacco smoking was significantly associated with LBP amongst the respondents. This was in agreement with previous studies in which the prevalence of LBP was higher amongst current smokers and ex-smokers than in non-smokers,^8^ as well as the study by Vindigni that reported higher levels of smoking in people with LBP.^11^

For the female respondents in this study, logistic regression analysis showed that a waist circumference of 88 cm or more (*p* = 0.005, OR = 1.029, CI = 1.009 – 1.050) was significantly associated with LBP. This is similar to the report in which 71% of the respondents were found to be either overweight or obese, with 16% not being physically active and 35.9% engaging in less than 30 minutes of exercise daily.^11^

Being overweight is more common in women compared with men. Its prevalence increases in mid-life and then decreases over time. Being overweight causes an increase in the pressure on the structures of the lower back and that may lead to lumbar disc herniation and subsequent LBP. Studies reported an association between being overweight and experiencing LBP.^7, 20^ Chronic medical illnesses, such as hypertension, diabetes mellitus and obesity with advancing age, have been reported to influence the occurrence of tendon and ligament diseases which can lead to LBP.^21^ For the female respondents, logistic regression analysis showed that dysmenorrhea (*p* = 0.012, OR = 2.260, CI = 1.197 – 4.268) was associated with LBP. This is similar to the study in which LBP and headaches were found to contribute the most to the severity of dysmenorrhoea.^22^

## Conclusion

Back pain is a common cause of absenteeism at work and functional disability, which has assumed a public health importance.^23^ In Australia, LBP is commonly treated with exercise.^11^ Lifestyle modification, such as regular exercise, smoking cessation and weight reduction, and culturally acceptable health promotion programmes have been found to be beneficial in reducing the disability associated with LBP.^5, 11^ Workers at risk of LBP need to modify their postures during occupational activities by preventing excessive flexion and extension movements.^2^ In our setting there is a high prevalence of LBP amongst adult patients, with several modifiable risk factors identified. Various levels of prevention should be employed to alter occupational activities and lifestyle habits.
